# Identification and Characterization of 40 Isolated *Rehmannia glutinosa* MYB Family Genes and Their Expression Profiles in Response to Shading and Continuous Cropping

**DOI:** 10.3390/ijms160715009

**Published:** 2015-07-02

**Authors:** Fengqing Wang, Yanfei Suo, He Wei, Mingjie Li, Caixia Xie, Lina Wang, Xinjian Chen, Zhongyi Zhang

**Affiliations:** 1 College of Agronomy, Henan Agricultural University, Zhengzhou 450002, China; E-Mails: heauzycxw@126.com (F.W.); suoyanfei1990@163.com (Y.S.); chenxinjian3978@126.com (X.C.); 2 Institute of Industrial Crops, Henan Academy of Agricultural Science, Zhengzhou 450002, China; E-Mail: weihezj123@126.com; 3 College of Crop Sciences, Fujian Agriculture and Forestry University, Fuzhou 350002, China; E-Mail: xinyuzszj@163.com; 4 School of medicine, Henan University of Traditional Chinese Medicine, Zhengzhou 450046, China; E-Mails: nanyangxcx@126.com (C.X.); lnsdhz2012@126.com (L.W.)

**Keywords:** MYB transcription factor, shading, continuous cropping, *Rehmannia glutinosa*

## Abstract

The v-myb avian myeloblastosis viral oncogene homolog (MYB) superfamily constitutes one of the most abundant groups of transcription factors (TFs) described in plants. To date, little is known about the MYB genes in *Rehmannia glutinosa*. Forty unique MYB genes with full-length cDNA sequences were isolated. These 40 genes were grouped into five categories, one R1R2R3-MYB, four TRFL MYBs, four SMH MYBs, 25 R2R3-MYBs, and six MYB-related members. The MYB DNA-binding domain (DBD) sequence composition was conserved among proteins of the same subgroup. As expected, most of the closely related members in the phylogenetic tree exhibited common motifs. Additionally, the gene structure and motifs of the *R. glutinosa* MYB genes were analyzed. MYB gene expression was analyzed in the leaf and the tuberous root under two abiotic stress conditions. Expression profiles showed that most *R. glutinosa* MYB genes were expressed in the leaf and the tuberous root, suggesting that MYB genes are involved in various physiological and developmental processes in *R. glutinosa*. Seven MYB genes were up-regulated in response to shading in at least one tissue. Two MYB genes showed increased expression and 13 MYB genes showed decreased expression in the tuberous root under continuous cropping. This investigation is the first comprehensive study of the MYB gene family in *R. glutinosa*.

## 1. Introduction

MYB transcription factors (TFs), a group of TFs with conserved DNA binding domains, are widely distributed in all eukaryotic organisms and constitute one of the largest TF families in the plant kingdom. MYB TFs contain a conserved DNA-binding domain (DBD) that is homologous to the DBD of animal c-Myb [1]. This domain generally consists of 1–4 imperfect amino acid sequence repeats (R0, R1, R2, and R3) of approximately 52 amino acids, each encoding three α-helices [2]. The second and third helices form a helix-turn-helix (HTH) structure [3] that intercalates in the major groove when bound to DNA. The third α-helix is thought to play a recognition role in binding to a short DNA sequence [4]. MYB proteins can be classified into 4R-MYB (contain four R1/R2-like repeats), R1R2R3-type MYB (3R-MYB), R2R3-MYB and MYB-related (contain single or a partial MYB repeat) depending on the number of adjacent repeats in the MYB domain (one, two, three or four) [2].

The first identified gene encoding a MYB domain protein in plants was *COLORED1* (*C1*), which is involved in anthocyanin biosynthesis in the aleurone layer of *Zea mays* [5]. An increasing number of plant MYB TF members have been identified and characterized in numerous plant species. Most plant MYB genes encode R2R3-MYB class proteins, which contain two repeats [2,6]. These proteins are thought to have evolved from an R1R2R3-MYB ancestor by the loss of the sequences encoding the R1 repeat and subsequent expansion of the family [7,8]. The diverse functions of these genes in plant-specific processes include primary metabolism [9,10,11,12], secondary metabolism [13,14,15,16], cell fate and identity [17,18,19,20,21], environmental stresses [22,23,24,25], and organ development [26,27,28,29,30,31,32].

*Rehmannia glutinosa*, one of the most famous Four Huai medicines, has been widely used as a medicinal herb with a long history of cultivation and application in China. The *R. glutinosa* genome has not yet been sequenced. There are only limited reports on the characterization of protein coding genes and non-coding miRNAs in *R. glutinosa* [33,34,35,36]. In contrast with the intensive research on MYB TFs in both model and crop plants such as *Arabidopsis thaliana*, *Oryza sativa*, *Glycine max* and *Z. mays* [2,37,38,39], there is no reported characterization of the MYB TFs in *R*. *glutinosa*. In this study, we identified a comprehensive set of MYB family members in *R. glutinosa*. Phylogenies, secondary protein structures, and gene expression patterns were analyzed and compared between conventional and novel classes of MYB candidates. These results will provide valuable information for understanding the classification and putative functions of *R. glutinosa* MYBs and other TF families and increase our understanding of potential transcriptional regulatory mechanisms in the development of the *R. glutinosa* tuberous root.

## 2. Results and Discussion

### 2.1. Identification of the MYB Gene Family in R. glutinosa

To predict *R. glutinosa* MYB genes at the transcriptome level, we downloaded all 197 *Arabidopsis* MYB cDNA sequences from GenBank (http://www.ncbi.nlm.nih.gov/genbank). BLASTn analysis of *Arabidopsis* MYBs against the current transcriptome database of *R. glutinosa* [35] was then performed using the BLASTn algorithm [40]. An *e*-value cut-off of 1 × 10^−5^ was applied. As a result, a total of 165 MYB unigenes of *R. glutinosa* were identified. Among the 165 unigenes, the lengths of 56 (about one third) sequences were between 200 and 500 bp. The other 109 sequences were longer than 500 bp. Forty sequences possessed complete opening reading frames (ORF) in ORF Finder Table 1 and Table S1. The remaining *R. glutinosa* MYB genes possessing incomplete ORFs were excluded from further analysis.

To validate the *MYB* candidates obtained using BLASTn, the proteins encoded by the 40 matching unigenes were analyzed using the online Batch CD-search tool (CDD, in NCBI) [41] and the SMART database [42]. Based on these results, all 40 MYB genes were confirmed to be MYB candidates by at least one of these two tools.

We also searched the NCBI database for plant kingdoms with identified MYB protein amino acid sequences and found that thirty-six sequences showed high identity (52%–91%) with *Sesamum indicum*, which belongs to the family *Pedaliaceae*. The other three genes, *RgMYB2*, *RgMYB3* and *RgMYB18*, matched to *Salvia miltiorrhiza*, which belong to the family *Labiatae* (Table 1). Another gene, *RgMYB6*, showed the highest identity with *Scutellaria baicalensis* (*Labiatae*). The three families *Pedaliaceae*, *Labiatae* and *Scrophulariaceae* belong to *Tubiflorae Sympetala*.

### 2.2. Protein Motif and Structure Analysis of the MYBs in R. glutinosa

**Figure 1:**
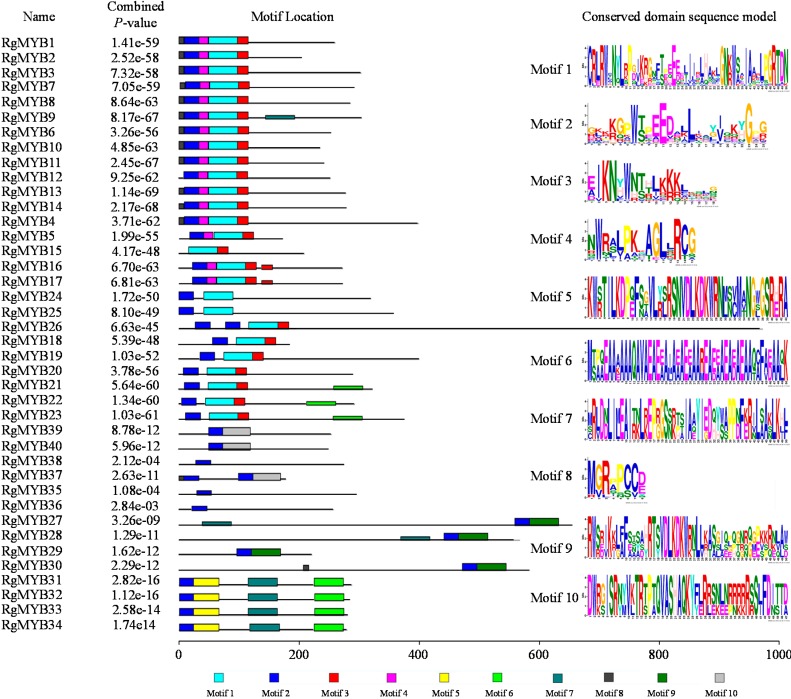
Schematic representation of conserved motifs in the MYB proteins of *R. glutinosa*, which were elucidated by MEME. Each motif is represented by a number in the colored box. The black lines represent the nonconserved sequences. The scale bar represents 200 amino acids.

To further reveal the divergence of the *R. glutinosa* MYB proteins, putative motifs were predicted by the program multiple em for motif elicitation (MEME), and 10 distinct motifs were identified. The positions of the MYB/SANT domains and any conserved motifs are shown in Figure 1. Motif 2 was found in all 40 MYB proteins. Simple Modular Architecture Research Tool (SMART) analysis revealed that seven motifs (motif 1, 2, 3, 4, 5, 9 and 10) are conserved MYB DNA-binding domains (DBD), motif 6 is a coiled coil region, motif 7 is a H15 domain, and motif 8 is an unknown function domain.

**Table 1:** MYB genes identified in *Rehmannia glutinosa* transcriptome.

Gene Name	Gene ID	Gene Length/bp	Amino Acid Length	Blast Results (Query Cover, *e*-Value, Identities, Accession No., Description, (Species))
*RgMYB1*	CL429Contig1	1126	273	95%, 2 × 10^−144^, 76%, XP_011070362.1, transcription repressor MYB5-like (*Sesamum indicum*)
*RgMYB2*	CL8750Contig1	831	210	90%, 2 × 10^−95^, 73%, AGN52078.1, MYB-related transcription factor (*Salvia* *miltiorrhiza*)
*RgMYB3*	Unigene7708	1036	318	100%, 0.0, 79%, AGN52106.1, MYB-related transcription factor (*S. miltiorrhiza*)
*RgMYB4*	CL128Contig2	1582	418	100%, 0.0, 70%, XP_011087570.1, transcription factor MYB86-like (*S. indicum*)
*RgMYB5*	Unigene19474	673	199	95%, 2 × 10^−89^, 72%, XP_011079754.1, transcription factor MYB114-like (*S. indicum*)
*RgMYB6*	Unigene18939	897	267	97%, 1 × 10^−96^, 59%, AGZ16400.1 MYB7 (*Scutellaria* *baicalensis*)
*RgMYB7*	CL7757Contig1	1207	306	100%, 8 × 10^−118^, 61%, XP_011086359.1, MYB-related protein Zm1 (*S. indicum*)
*RgMYB8*	Unigene14277	1100	299	100%, 4 × 10^−161^, 73%, XP_011098622.1, MYB-related protein 306-like (*S. indicum*)
*RgMYB9*	Unigene7159	1338	319	100%, 1 × 10^−153^, 74%, XP_011071639.1, MYB-related protein 306-like (*S. indicum*)
*RgMYB10*	Unigene16684	1017	247	100%, 4 × 10^−92^, 63%, XP_011080041.1, MYB-related protein Myb4-like (*S. indicum*)
*RgMYB11*	Unigene9736	1347	251	100%, 4 × 10^−149^, 83%, XP_011091315.1, MYB-related protein Myb4-like (*S. indicum*)
*RgMYB12*	Unigene12270	862	265	99%, 3 × 10^−71^, 52%, XP_011091328.1, MYB-related protein Myb4-like (*S. indicum*)
*RgMYB13*	Unigene12354	1307	292	97%, 5 × 10^−95^, 56%, XP_011100326.1, MYB-related protein Myb4-like (*S. indicum*)
*RgMYB14*	Unigene21429	1227	293	98%, 1 × 10^−105^, 59%, XP_011100326.1, MYB-related protein Myb4-like (*S. indicum*)
*RgMYB15*	CL5174Contig3	1101	219	94%, 2 × 10^−84^, 68%, XP_011089822.1, transcription factor MYB48-like (*S. indicum*)
*RgMYB16*	CL9333Contig1	1327	286	100%, 4 × 10^−139^, 75%, XP_011076599.1, transcription factor MYB108-like (*S. indicum*)
*RgMYB17*	Unigene5899	1217	288	96%, 1 × 10^−131^, 74%, XP_011070788.1, transcription factor MYB24 (*S. indicum*)
*RgMYB18*	CL6949Contig1	790	202	91%, 8 × 10^−94^, 74%, AGN52041.1, MYB-related transcription factor (*S. miltiorrhiza*)
*RgMYB26*	CL3700Contig2	3518	1015	100%, 0.0, 75%, XP_011081822.1, MYB-related protein 3R-1 (*S. indicum*)
*RgMYB19*	Unigene19140	1451	419	100%, 3 × 10^−155^, 55%, XP_011076754.1, uncharacterized protein (*S. indicum*)
*RgMYB20*	Unigene9854	1257	303	100%, 2 × 10^−116^, 62%, XP_011088207.1, transcription factor MYB44-like (*S. indicum*)
*RgMYB21*	CL9364Contig2	1570	338	100%, 4 × 10^−162^, 74%, XP_011074411.1, transcription factor MYB44-like (*S. indicum*)
*RgMYB22*	CL6089Contig2	1303	307	86%, 1 × 10^−142^, 70%, XP_011099313.1, transcription factor MYB44-like (*S. indicum*)
*RgMYB23*	Unigene11755	1536	374	84%, 6 × 10^−161^, 80%, XP_011099313.1, transcription factor MYB44-like (*S. indicum*)
*RgMYB24*	CL173Contig2	1384	335	100%, 0.0, 79%, XP_011077649.1, transcription factor AS1 (*S. indicum*)
*RgMYB25*	Unigene5357	1478	387	96%, 0.0, 84%, XP_011097206.1, protein rough sheath 2 homolog (*S. indicum*)
*RgMYB27*	CL5870Contig1	2525	697	99%, 0.0, 74%, XP_011085026.1, telomere repeat-binding protein 4 (*S. indicum*)
*RgMYB28*	CL7811Contig2	1869	593	100%, 0.0, 73%, XP_011094643.1, uncharacterized protein (*S. indicum*)
*RgMYB29*	Unigene9541	836	233	100%, 3 × 10^−105^, 69%, XP_011094644.1, uncharacterized protein (*S. indicum*)
*RgMYB30*	Unigene18531	2173	616	100%, 0.0, 73%, XP_011079786.1, uncharacterized protein (*S. indicum*)
*RgMYB31*	CL4303Contig4	1484	302	100%, 8 × 10^−161^, 90%, XP_011088080.1, telomere repeat-binding factor 1 (*S. indicum*)
*RgMYB32*	Unigene21243	1440	300	100%, 2 × 10^−145^, 84%, XP_011088080.1, telomere repeat-binding factor 1 (*S. indicum*)
*RgMYB33*	CL4502Contig2	1359	295	100%, 2 × 10^−126^, 75%, XP_011099389.1, telomere repeat-binding factor 1 (*S. indicum*)
*RgMYB34*	CL4502Contig3	1226	294	100%, 8 × 10^−138^, 82%, XP_011099389.1 telomere repeat-binding factor 1 (S. indicum)
*RgMYB35*	CL3772Contig3	1175	328	100%, 3 × 10^−176^, 73%, XP_011095766.1, MYB family transcription factor APL (*S. indicum*)
*RgMYB36*	Unigene13894	1053	272	68%, 3 × 10^−111^, 91%, XP_011092595.1, MYB family transcription factor APL (*S. indicum*)
*RgMYB37*	CL3105Contig2	714	187	100%, 5 × 10^−103^, 82%, XP_011097737.1, transcription factor Divaricata-like (*S. indicum*)
*RgMYB38*	CL725Contig2	1561	288	100%, 5 × 10^−177^, 88%, XP_011094729.1, protein REVEILLE 8 (*S. indicum*)
*RgMYB39*	CL5749Contig2	1086	266	100%, 6 × 10^−133^, 83%, XP_011071772.1, transcription factor MYB1R1 (*S. indicum*)
*RgMYB40*	CL613Contig1	1138	260	100%, 2 × 10^−111^, 72%, XP_011071772.1, transcription factor MYB1R1 (*S. indicum*)

**Figure 2:**
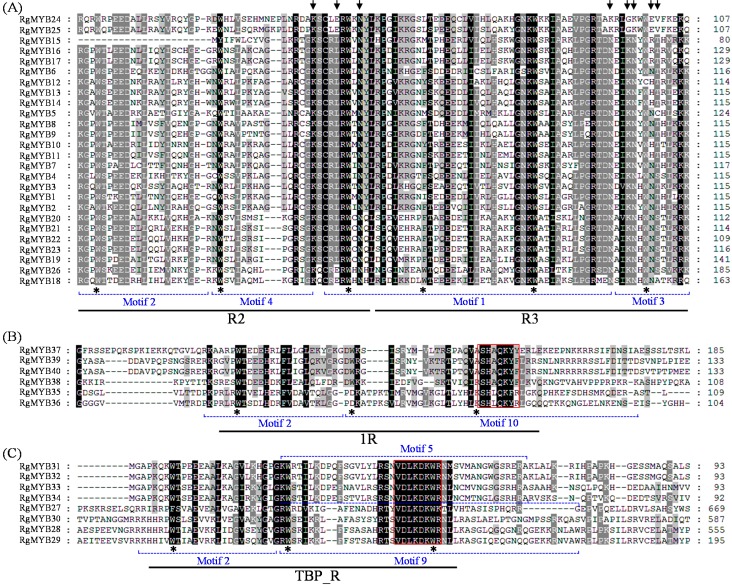
Highly conserved amino acid residues are present among the R2R3 (**A**), 1R (**B**) and TBP_R (**C**) domains of the *R. glutinosa* MYBs. The three regularly spaced Trp residues present in each repeat of the MYBs are labeled with asterisks. The amino acid sequences are aligned, and gaps (dots) have been introduced to maximize the alignment. The shading of the alignment presents identical residues in black, conserved residues in dark gray, and similar residues in light gray. The positions corresponding to base-contacting residues of the human MYBs are marked with arrows. The highly conserved SHAQKYF motifs in 1R and VDLKDKWR motif in TBP_R are boxed. Position of motif 2 plus motif 4 and part of motif 1 in Figure 1 relative to the R2 domain, motif 1 plus motif 3 relative to the R3 domain, motif 2 plus motif 10 relative to 1 R domain, motif 2 plus motif 5 or motif 9 relative to TBP_R domain.

Multiple amino acid sequence alignments using the BLASTp program and Clustal Omega showed that the 40 *R. glutinosa* MYBs were separated into three groups based on sequence similarity of the conserved MYB domain (Figure S1). Further sequence alignment revealed that the 26 proteins of group I shared the R2 and R3 MYB repeats and contained characteristic amino acids, including a series of evenly distributed and highly conserved Trp (W) residues (Figure 2A). As shown in Figure 2, these Trp (W) residues, except the first Trp (W) residues of the R3 repeat, are highly conserved in *R. glutinosa* MYB DBDs. In addition, RgMYB26 has another MYB repeat belonging to the R1R2R3 MYB protein. The six proteins of group II shared a MYB domain similar to the second MYB repeat of tomato LeMYBI [43], which acts as a transcriptional activator in yeast and plants and contains a SHAQKYF amino acid signature motif in the MYB-like repeat (Figure 2B). The sequence alignment of group III proteins indicated that eight MYBs contained a MYB-containing region of TRFL proteins that bound to the human telomeric DNA repeat sequence [44]. The most highly conserved region within all of the TRFL proteins, VDLKDKWR, lies within the telobox consensus sequence (Figure 2C).

To learn more about the structures of the MYB genes, we performed protein homology modeling on three *R. glutinosa* MYB proteins using SWISS-MODEL. We used the structure of the *A. thaliana* MYB proteins as templates. A typical MYB domain is composed of a three-α-helix bundle, which is thought to participate in binding DNA [45]. The generated molecular model showed that RgMYB40, RgMYB1, and RgMYB26 have three, six and nine α-helices, respectively (Figure S2).

### 2.3. Phylogenetic Analysis of the MYB Proteins

**Figure 3:**
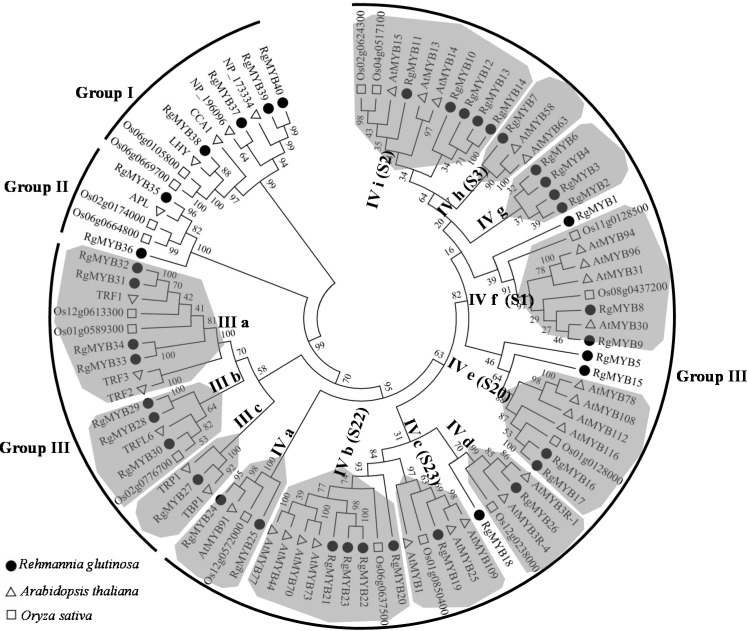
Phylogenetic tree of the MYB transcription factors from *R. glutinosa*, *Arabidopsis* and rice. The full-length sequences of the MYB proteins were aligned using MAFFT, and the phylogenetic tree was constructed using the neighbor-joining method in the MEGA6 software [46] (Tamura *et al.*, 2013). Bootstrap values (shown at the corresponding nodes) were obtained from 1000 replicates and are reported as percentages. The colored shadow marks the subgroups of the MYBs.

To further examine the evolutionary relationship among *R. glutinosa*, *Arabidopsis* and rice MYB proteins, the complete amino acid sequences of all MYB proteins were subjected to a multiple sequence alignment using the MAFFT program. The multiple sequence alignment file was then used to construct an unrooted phylogenetic tree using MEGA6.06 [46] by employing the neighbor-joining method. As shown in Figure 3, the phylogenetic tree divided the MYBs into four groups (groups I through IV). Group IV constitutes the largest clade with 58 MYBs, followed by group III (17 proteins). In addition, ten and five MYBs fall into groups I and II, respectively.

As expected, most of the closely related members in the phylogenetic tree exhibited common motif compositions, suggesting that there are functional similarities among the MYB proteins within the same group. Specifically, Six RgMYBs (RgMYB37, 38, 39, 40, 35 and 36) were assembled together with LHY and APL [47,48] in groups I and II, which represent the functional group of the regulation of circadian clock or phloem identity. Among them, RgMYB37 is a 2R MYB protein with two DBDs that are separated by long linker sequences. The other five RgMYB proteins are 1R MYB proteins with only one MYB domain with a SHAQKYF motif. The four RgMYB genes of subgroup IIIb and IIIc encode proteins with a single MYB domain at the C-terminus, similar to human TRF1 and *Arabidopsis* TBP1 [49,50]. The four RgMYB proteins of subgroup IIIa are members of the SMH (single MYB histone) protein family, which comprises double-stranded DNA-binding proteins that are specific to higher plants. They contain a MYB domain (motif 2 plus motif 5) at the N-terminus, a central H1/H5-like domain (motif 7) and a C-terminally located coiled-coil domain (motif 6). The SMH protein may specifically interact through the MYB domain with telomeric double-stranded DNA *in vitro*, while the central H1/H5-like domain interacts non-specifically with DNA sequences and mediates protein–protein interactions [51,52].

All the group IV proteins contain two repeats (R2R3), which are thought to have evolved from an *R1R2R3-MYB* gene ancestor by the loss of the sequences encoding the R1 repeat and subsequent expansion of the gene family [8]. Group IV could be further divided into nine subgroup (named Iva–IVi). Similar to previous reports, the MYB genes with the same functions exhibited a tendency to cluster into one subgroup. For instance, subgroup IVb encompassed four *R. glutinosa* proteins homologous to AtMYB73/44 (*Arabidopsis* subgroup 22, S22), which regulates ABA-mediated stomatal closure in response to abiotic stresses [23]. Two proteins of subgroup IVa, RgMYB24 and RgMYB25, showed the highest sequence identity with AS1 and rough sheath 2 of *S. indicum*, respectively. AS1 and rough sheath 2 are orthologous genes from *Arabidopsis* and maize with similar molecular functions [53]. Two RgMYB proteins of subgroup IVe showed the highest identification with subgroup 20 of *Arabidopsis*, which is implicated in stress responses [22,24]. The five *R. glutinosa* proteins of subgroups IVi had high identity with subgroup 2 of *Arabidopsis*. The 3R-MYB protein, RgMYB26, was grouped into IVd with two *Arabidopsis* 3R-MYB proteins AtMYB3R-1 and AtMYB3R-4 [54], representing the functional group with proteins responsible for regulation of cytokinesis.

### 2.4. Differential Expression of R. glutinosa MYB Genes in Various Tissues/Developmental Stages

**Figure 4:**
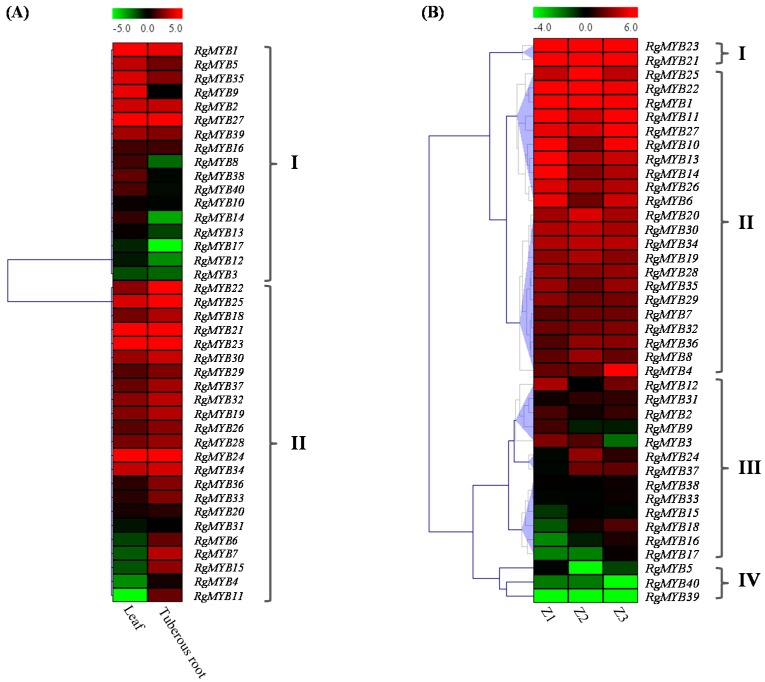
Expression profiles of 40 *R. glutinosa* MYB family genes in different tissues/developments. (**A**) Expression levels of 40 MYBs in leaves and tuberous roots; (**B**) Expression levels of 40 MYB genes in tuberous roots at different stages. The color bar presented expression level of genes: green shows the low expression, and red color shows high expression level. Z1, Seedling stage; Z2, Elongation stage of tuberous root; and Z3, Pre-expanding stage of tuberous root. The heat maps were generated by MultiExperiment Viewer (MeV).

The gene expression pattern can provide important clues to gene function. We therefore analyzed the expression of *R. glutinosa* MYB genes in leaf and tuberous root by RNA-Seq. The expression profiles of all MYB family genes in *R. glutinosa* leaves and roots were evaluated using Pearson correlation Hierarchical Clustering with MultiExperiment Viewer (MeV) 4.9 software [55]. The results showed that 40 MYB genes were divided into two categories (Figure 4A and Table S2). Seventeen of the MYB genes were in group I and showed higher expression in leaves than in tuberous roots. The other 23 MYB genes formed group II and were highly expressed in tuberous roots. Among of them, eight MYB genes, including *RgMYB4*, *RgMYB6*, *RgMYB7*, *RgMYB11*, *RgMYB15*, *RgMYB22*, *RgMYB25* and *RgMYB33*, were differently expressed genes.

To further study the potential roles of MYB genes in the formation of *R. glutinosa* tuberous roots, the expression profiles of 40 MYB genes in tuberous roots at the seedling stage (Z1), elongation and pre-expansion of tuberous root (Z2 and Z3) were evaluated using Euclidean distance Hierarchical Clustering with MeV 4.9 software. The results showed that 40 MYB genes fell into four groups (Figure 4B and Table S3). In particular, the expression levels of two MYB genes in group I, *RgMYB23* and *RgMYB21*, were high in tuberous roots at all three stages. In contrast, the expression levels of three MYB genes, *RgMYB5*, *RgMYB40* and *RgMYB39*, were very low during tuberous root development. Twenty-two MYB genes of group II showed high expression levels with minor differences, similar to constitutive expression. More than half of the 13 MYB genes in group III exhibited differential expression at different development stages of tuberous roots. In addition, K-means clustering was conducted to analyze the co-expression, and 40 MYB genes were divided into seven clusters based on their expression modulation (Figure S3). These clusters reflected the general trends and key transitional states during tuberous root development. Group I contains genes that first increased in expression at the Z2 stage and then decreased at the Z3 stage, while genes in group VII showed the opposite tendency. The highest expression levels of genes in group II and V were at the Z1 and Z3 stages, respectively. However, the transcriptional abundance of genes in groups III and VI were the lowest at the Z3 and Z1 stages, respectively.

### 2.5. Expression Profiling of MYB Genes under Shading and Continuous Cropping Stresses

**Figure 5:**
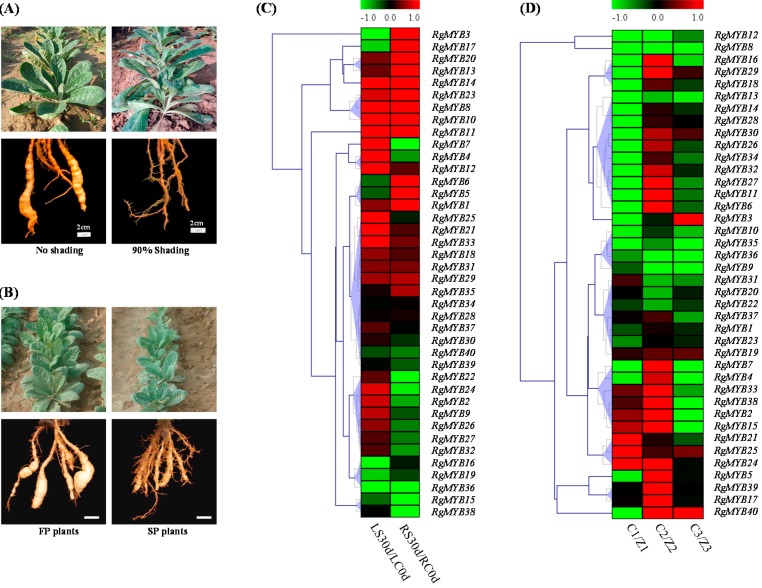
Growth of *R. glutinosa* seeding and expression profiles of *R. glutinosa* MYB genes under abiotic stresses. (**A**) Effect of shading stress in *R. glutinosa* seedlings. Thirty-day-old seedlings were shaded and grown for an additional 35 days; (**B**) Phenotypes of 90-day-old *R. glutinosa* plants under continuous cropping conditions; White bars: 2 cm; (**C**) Heat map showing the signal differences of 40 MYB genes in leaf and tuberous root under 90% shading compared to control; (**D**) Heat map showing the signal intensities of 40 MYB transcripts in tuberous roots at Z1, Z2 and Z3 stage under continuous cropping. The color bar presented expression level of genes: green shows the low expression, and red color shows high expression level. Z1, Seedling stage; Z2, Elongation stage of tuberous root; and Z3, Pre-expanding stage of tuberous root. The heat maps were generated by MultiExperiment Viewer (MeV).

*R. glutinosa* is a light-demanding plant species. Under shading conditions, development of *R. glutinosa* seedlings was inhibited, and the plants displayed decreased sizes of leaves and tuberous roots (Figure 5A). To identify MYB genes with a potential role in the shading response of plants, we analyzed the expression pattern of *R. glutinosa* MYB genes in response to shading. The expression of MYB genes was examined in the leaf and tuberous root under shading conditions by RNA-Seq. It was found that 70% (28) and 52.5% (21) of MYB genes were highly expressed to varying degrees in 90% shaded leaves and tuberous roots, respectively (Figure 5C and Table S4). Hierarchical clustering divided the 40 MYB genes into four groups based on their expression profiles in response to shading. Four and 11 MYB genes were differentially expressed in leaves and tuberous roots, respectively, after shading treatment. Among these genes, two MYB genes (*RgMYB10* and *RgMYB14*) were strongly up regulated in both leaves and tuberous roots. Interestingly, under shading treatment, eight MYB genes (*RgMYB1*, *RgMYB3*, *RgMYB10*, *RgMYB11*, *RgMYB13*, *RgMYB14*, *RgMYB20* and *RgMYB23*) were apparently induced, but only three genes (*RgMYB7*, *RgMYB15* and *RgMYB22*) were repressed. The qRT-PCR results agree with the results of the RNA-seq experiment (Figure S4).

Continuously cropped *R. glutinosa* plants suffer from severe problems, including reduced biomass and decreased tuberous root products [35]. Continuous cropping significantly inhibited the growth of *R. glutinosa*, as indicated by reduced growth width, number of leaves per plant, leaf length, leaf width, tuberous root length, tuberous root width and tuberous fresh weight after two years of continuous cropping (Figure 5B). With the goal of identifying candidate continuous cropping responsive MYB genes, we analyzed the expression levels of 40 MYB genes from RNA-Seq data. There were 27 (67.5%) MYB genes down-regulated in tuberous roots at the Z1 stage, and 12 (*RgMYB6*, *RgMYB8*, *RgMYB11*, *RgMYB12*, *RgMYB13*, *RgMYB14*, *RgMYB26*, *RgMYB27*, *RgMYB28*, *RgMYB29*, *RgMYB30* and *RgMYB34*) were differentially expressed genes (Figure 5D and Table S5). Curiously, unlike at the Z1 stage, up to 28 (70%) MYB genes were up-regulated at the Z2 stage, but only one was differentially expressed. Interestingly, more than 80% of MYB genes were down-regulated at the Z3 stage. This result suggests that the response of MYB genes to the continuous cropping of *R. glutinosa* mainly occurred at the Z2 stage.

### 2.6. Discussion

In plants, MYB factors are one of the largest TF families [2,56]. More than 20 years ago, the first gene encoding a MYB TF in plants was identified. With the growing number of fully sequenced plant genomes, the identification of MYB genes has increased in recent times. Genome-wide analysis led to the identification of 155, 197 and 75 MYB genes in rice, *Arabidopsis* and sugar beet, respectively, which map to different chromosomes [57,58]. *R2R3-MYB* gene families have been annotated genome-wide in *A. thaliana* (126 members) [3], *Z. mays* (157 members) [59], *O.* *sativa* (102 members) [60], *Vitis* *vinifera* (117 members) [61], *Populus* *trichocarpa* (192 members) [62], *G.* *max* (244 members) [38] and *Malus* × *domestica* (222 members) [63]. However, no large dataset of MYB TFs is available for *R. glutinosa*.

In the present study, we identified 40 MYB genes with complete ORFs from *R. glutinosa*. Ten motifs were predicted by MEME, and seven of them were MYB/SANT domains according to SMART analysis. However, these motifs did not contain the basic features of whole MYB DBD domains. It may be that there were less well conserved DBD domain sequences among the diverse subgroup of *R. glutinosa* MYB proteins. Multiple amino acid sequence alignments using the BLASTp program and Clustal Omega showed that the 40 MYBs were separated into three groups based on sequence similarity of conserved MYB domain (Figure S5). Conserved domains of the three groups of MYB proteins were predicted by GLAM2 [64], and full-length MYB DBDs were identified that contained typical structure sequences. This result suggested that GLAM2 might even be used to analyze the domains of proteins in the same subfamily.

The six RgMYB proteins of groups I and II shared a MYB domain similar to the second MYB repeat of tomato LeMYBI [43], which acts as a transcriptional activator in yeast and plants. It contains a SHAQKYF amino acid signature motif in the MYB-like repeat (Figure 2B). A comparison of amino acid residues between the 1R regions of the six MYBs showed that the tryptophan residue is conserved at the first and second positions but not at the third position among most of the 1R regions. The sequence alignment of group III proteins indicated that the eight MYBs contained a MYB-containing region of TRFL proteins that bound to the human telomeric DNA repeat sequence [44]. The most highly conserved region within all of the TRFL proteins, VDLKDKWR [50], lies within the telobox consensus sequence (Figure 2C). We also observed that the third helix is better conserved than the first two helices in the DBD domain. It was previously demonstrated that the residues in the third helix are important for DNA binding by interacting with the DNA bases in the major groove when bound to DNA [65]. Further sequence alignment revealed that the 26 RgMYB proteins of group IV shared the R2 and R3 MYB repeats and contained characteristic amino acids, including a series of evenly distributed and highly conserved Trp (W) residues (Figure 2A) that are known to play key roles in sequence-specific DNA binding [38,66] and serve as landmarks of plant MYB proteins. As shown in Figure 2, these Trp (W) residues, except the first Trp (W) residue of the R3 repeat, are highly conserved in *R. glutinosa* MYB DBDs. In addition, RgMYB26 also has another MYB repeat, belonging to the R1R2R3 MYB protein.

To learn more about the structures of the MYB genes, we performed protein homology modeling on three MYB proteins of *R. glutinosa* using SWISS-MODEL. We used the solved structure of *A. thaliana* MYB proteins as templates. The typical MYB domain is comprised of a three-α-helix bundle that is thought to bind DNA [45]. The generated molecular model showed that RgMYB40, RgMYB1, and RgMYB26 have three, six and nine α-helices, respectively (Figure S2).

The topology of the MYB phylogeny indicates that some MYB genes in the same subgroup of *Arabidopsis* have the same function and that some MYB genes with similar functions are located in the same subgroup [67]. For instance, AtMYB11/PFG1, AtMYB12/PFG1 and AtMYB111/PFG3 (subgroup 7) control flavonol biosynthesis in all *Arabidopsis* tissues [14]. Similarly, AtMYB75, AtMYB90, AtMYB113 and AtMYB114 (subgroup 6) control anthocyanin biosynthesis in *Arabidopsis* vegetative tissues [15]. The R2R3-MYB proteins of *Arabidopsis* subgroup 12 regulate glucosinolate biosynthesis, and AtMYB28, AtMYB29 and AtMYB76 regulate the biosynthesis of aliphatic glucosinolates in aerial issues [10,15]. In this report, some *R. glutinosa* MYB proteins fell into a subgroup (Figure 3) that showed the highest homology to the *Arabidopsis* MYBs of another subgroup. Moreover, functions are broadly conserved for the MYB proteins of the same subgroup in different angiosperms. For example, two MYB orthologs, the snapdragon *PHAN* gene and the maize *rs2* gene, are located in subgroup G18. Both genes are involved in organ development [68,69]. *R. glutinosa* MYB proteins may share similar functions to *Arabidopsis* homologs in the same phylogenetic clades.

Functional studies on R2R3-MYB revealed that MYB genes in the same subgroup not only possess similar functions but are also co-expressed after stress treatments [70]. For example, *AtMYB44*, *AtMYB77* and *AtMYB73* of *Arabidopsis* subgroup 12 were up-regulated by wounding [71] and were also found to be up-regulated in *sos2* mutants upon salt stress treatment [72]. The four MYB genes *AtMYB44*, *AtMYB70*, *AtMYB73* and *AtMYB77* showed similar temporal and spatial expression patterns and levels [70]. MYB genes in the same subgroup have redundant functions, as is supported by the co-expression phenomenon.

Most of the MYB genes in subgroup 2 are involved in *Arabidopsis* stress regulation [73]. In this study, five MYB genes (*RgMYB10*, *RgMYB11*, *RgMYB12*, *RgMYB13* and *RgMYB14*) in subgroup IVi (Figure 3), which are homologous to *Arabidopsis* MYB genes in subgroup 2, showed similar expression patterns during the development of tuberous roots and in response to continuous cropping (Figure 4B and Figure 5D). Most of them were differentially expressed under shading and continuous cropping conditions, implying that they could take part in the shading and continuous cropping responses in *R. glutinosa*.

Members of *Arabidopsis* subgroup 22 are involved not only in stresses response but also in plant development [28,73]. The expression levels of three orthologs of *AtMYB73*/*44* (*Arabidopsis* subgroup 22), *RgMYB20*, *RgMYB21* and *RgMYB23*, were up-regulated under shading conditions in leaf and/or tuberous root. However, only one gene (*RgMYB22*) was down regulated under shading. However, these genes were not differentially expressed under continuous cropping at the three *R. glutinosa* tuberous root stages. These genes may play important roles in the response to shading. *AtMYB59* regulates root development through the control of cell cycle progression at the root tips [30]. Interestingly, we also found that an ortholog of *AtMYB48/59* was up regulated in *R. glutinosa* tuberous root under shading and may also be involved in the shading response. Our results showed that 27 (67.5%), 12 (30%) and 33 (82.5%) MYB genes were down-regulated in *R. glutinosa* tuberous roots at the Z1, Z2 and Z3 stages, respectively. Most *R. glutinosa* MYB genes were up-regulated in tuberous roots at the Z2 stage, suggesting that the Z2 stage might be the critical point in the formation of continuous cropping problems in *R. glutinosa*. Therefore, how MYB family genes participate in shading or continuous cropping stresses are intriguing issues for further study.

## 3. Experimental Section

### 3.1. Plant Material and Sample Collection

Our experimental *R. glutinosa* plants, “Wen 85-5”, were grown in 2014 at Henan Agricultural University, Zhengzhou, China. The tuberous roots of *R. glutinosa* used for cultivation in all treatments were planted on 24 April 2014. Fields were maintained with locally normal production conditions. For shading experiments, more than 80 plants (four lines) were selected as untreated controls, while the other 80 plants were shaded to 10% of full sun with a high-density black-polypropylene shade cloth at 30 days after emergence. On 8 June (35 days after shading), leaves and tuberous roots of six randomly selected plants were collected from both control and treated seedlings. For the continuous cropping experiment, a group of seedlings was grown in a field in which *R. glutinosa* had not been planted for more than 10 years. The other group was grown in a field in which the same cultivar had been grown the previous year (planted on 25 April and harvested on 16 November, 2013). For convenience of description, we named the former group the first year (FP) planted *R. glutinosa* and the latter group as the second year (SP) replanted group. Each plant was regarded as a biological replicate. All samples mentioned above were immediately frozen in liquid nitrogen and stored at −80 °C until use.

### 3.2. Identification of Putative MYB mRNAs

Sequences derived from the *R. glutinosa* expressed sequence tag (EST) database, a collection of more than 87,665 *R. glutinosa* leaf and root unigenes (mean length 554 bp), were assembled using SOAPdenovo software (http://soap.genomics.org.cn/) [74]. Coding sequence (CDS) of MYB genes from *A. thaliana* were used to search for *R. glutinosa* homologs in the EST database using basic local alignment (BLASTn). To remove redundancy, the sequences were assembled using the SeqMan function of the DNAStar software package and adjusted manually. Only the sequences that shared >95% matches were considered redundant. The open reading frames (ORFs) were predicted using NCBI ORF Finder [75] and were translated into amino acid sequences. Finally, to confirm that the obtained sequences were MYB members, all of the non-redundant amino acid sequences of the primary identified MYB members were submitted to the website http://pfam.sanger.ac.uk to predict the MYB domains. Only the sequences that shared the MYB domain were confirmed to be MYB members. The *R. glutinosa* MYB sequences reported in this work have been submitted to GenBank and their accession numbers are KR780077-KR780116.

### 3.3. Protein Structure and Phylogenetic Analysis

To identify potential *R. glutinosa* MYB protein motifs, we used the MEME version 4.9.1 tool [76] with the following parameter settings: the distribution of motifs, 0 or 1 per sequence; maximum number of motifs to find, 10; minimum width of motif, 6; maximum width of motif, 50. In addition, only motifs with an *e*-value ≤1 × 10^−10^ were retained for further analysis. Subsequently, the SMART (http://smart.embl.de/) [42] program was used to search for detected motifs in protein databases. The 3-D MYB protein models were constructed using the SWISS-MODEL programs [77].

The amino acid sequences of *Arabidopsis* (2 R1R2R3-MYB, 21 R2R3-MYB, and 11 MYB-related members) and rice (1 R1R2R3-MYB, 8 R2R3-MYB, and 7 MYB-related members) MYB proteins were downloaded from the NCBI database. The complete amino acid sequences of MYB proteins were used to construct phylogenetic trees. Phylogenetic trees were constructed with MEGA6.06 (www.megasoftware.net) by the neighbor-joining method and bootstrap analysis (1000 replicates) [46].

### 3.4. Expression Profile Analysis of MYB Genes

The total RNA from the leaf and the tuberous root was extracted using TRIzol reagent (Invitrogen, Carlsbad, CA, USA) and treated with DNase I to degrade any possible DNA contamination. Subsequently, the mRNA was enriched using oligo(dT) magnetic beads. The cDNA fragments were enriched by PCR amplification and then purified by magnetic beads. The library products were sent to BGI-Shenzhen for sequencing via the Ion Proton platform. Dirty raw reads were discarded via the following three steps: (1) reads with adaptors were removed; (2) reads with nucleotides less than 30 were removed; (3) remaining reads were trimmed (15 nt from 3′ end); and (4) clean reads were obtained. The clean reads were mapped to the UniGene reference transcriptome using TRAP software [74]. Sequences with no more than 2 base pair mismatches were used in the alignment. The gene expression level was calculated using the RPKM (Reads Per Kilo bases per Million reads) method [78], and the formula is shown as follows:

(1) $$\text{RPKM}=\frac{10^{6}\text{C}}{\text{NL}/10^{3}}$$

where RPKM(A) is the expression level of gene A, C is the number of reads that uniquely aligned to gene A, N is the total number of reads that uniquely aligned to all genes, and L is the number of bases of gene A. The *p*-value corresponds to a differential gene expression test. FDR (False Discovery Rate) is a method to determine the *p*-value threshold in multiple tests. We used “FDR ≤ 0.001 and the absolute value of log_2_Ratio ≥ 1” as the threshold to judge the significance of the gene expression differences. More stringent criteria with smaller FDRs and larger fold-change values can be used to identify differentially expressed genes.

### 3.5. Quantitative Real-Time Polymerase Chain Reaction (PCR) Analysis

Total RNA was extracted from different *R. glutinosa* fresh samples. Approximately 50 mg tissue was collected and extracted using TRIzol reagent (Invitrogen). The RNA concentration was measured using a spectrophotometer, and integrity was ensured through analysis on a 1.5% (*w*/*v*) agarose gel. First-strand cDNA was synthesized in a 20 μL mixture containing 1 μg total RNA, 2 μL of 50 μM oligo-(dT)_12–18_ primers, 1 μL RNase inhibitor and 1 μL M-MLV reverse transcriptase (Invitrogen), incubated at 37 °C for 50 min, and then heated at 70 °C for 15 min.

Real-time quantitative reverse transcription PCR (qRT-PCR) was performed for mediator subunit genes on an iCycler thermal cycler (iQ5, Bio-Rad, Hercules, CA, USA) Real Time PCR instrument. The *RgTIP41* gene was used as endogenous control. The primers used are listed in Table S6. The PCR was conducted in a 25 μL mixture containing 12.5 μL SYBR^®^ Premix Ex Taq™ II (Tli RNaseH Plus) (Takara Bio, Dalian, China), 1 μL cDNA template, and 1 μL primers. The PCR program was: 95 °C for 30 s, followed by 40 cycles of 95 °C for 5 s and 58 °C for 30 s. Data were collected using the Bio-Rad iQ5 data detection system following the instructions. All reactions were performed in triplicate. After each assay, a dissociation kinetics analysis was performed to verify the specificity of the amplification products. Relative amounts of all mRNAs were calculated using the 2^−ΔΔ*C*t^ method [79]. The data for three biological replicates were analyzed using an analysis of variance (ANOVA) followed by Student’s *t*-test (*p* < 0.05).

## 4. Conclusions

In the present study, 40 MYB genes were identified in *R. glutinosa*. These 40 genes were grouped into five categories on the basis of their phylogenetic relationships, which were well supported by additional conserved protein motifs. The expressions of some of these MYB genes varied under shading and continuous cropping stresses, suggesting that MYBs may play conserved and various roles, indicating some MYBs may be involved in *R. glutinosa* tuberous root development and abiotic stresses. These observations contribute valuable information for understanding the roles of MYB genes in regulating *R. glutinosa* tolerance to shading and continuous cropping.

## Supplementary Materials

Supplementary materials can be found at http://www.mdpi.com/1422-0067/16/07/15009/s1.
