# Supplementary material for: Identification and Characterization of 40 Isolated Rehmannia glutinosa MYB Family Genes and Their Expression Profiles in Response to Shading and Continuous Cropping
Source: Int J Mol Sci. 2015 Jul 2;16(7):15009–30. doi: 10.3390/ijms160715009 (PMC4519885; doi:10.3390/ijms160715009)
Supplement: Supplementary file 1 [file ijms-16-15009-s001.zip › ijms-86807-supplementary-final/ijms-86807-supplementary-figures.pdf]

Supplementary Information

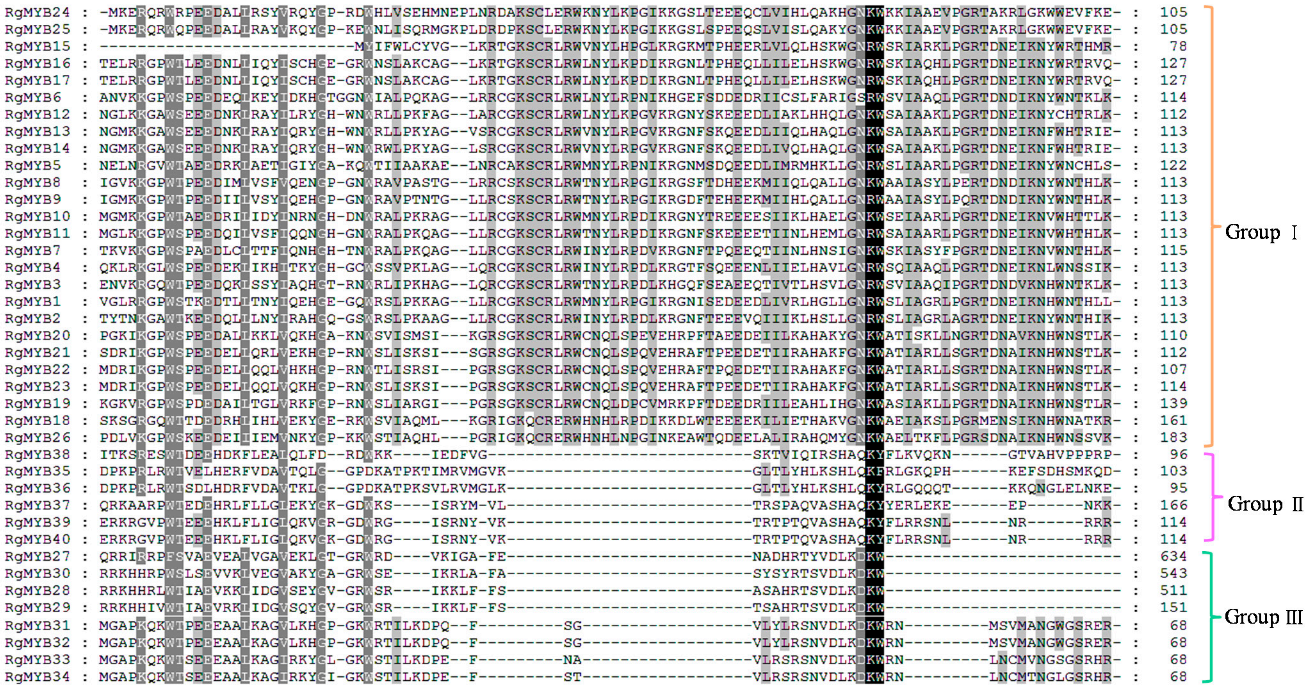

Figure S1. Conserved amino acid residues are present among the 40 proteins of the *R. glutinosa* MYBs. The amino acid sequences are aligned, and gaps (dots) have been introduced to maximize the alignment. The shading of the alignment presents identical residues in black, conserved residues in dark gray, and similar residues in light gray.

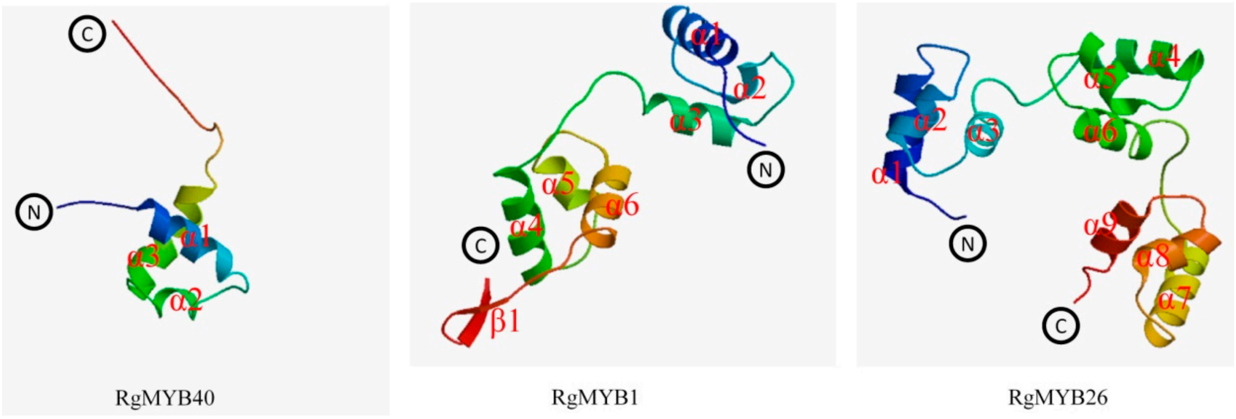

Figure S2. 3-D structure analysis of RgMYB transcription factors. C, C-terminal; N, N-terminal; α, α helix; β, β sheet.

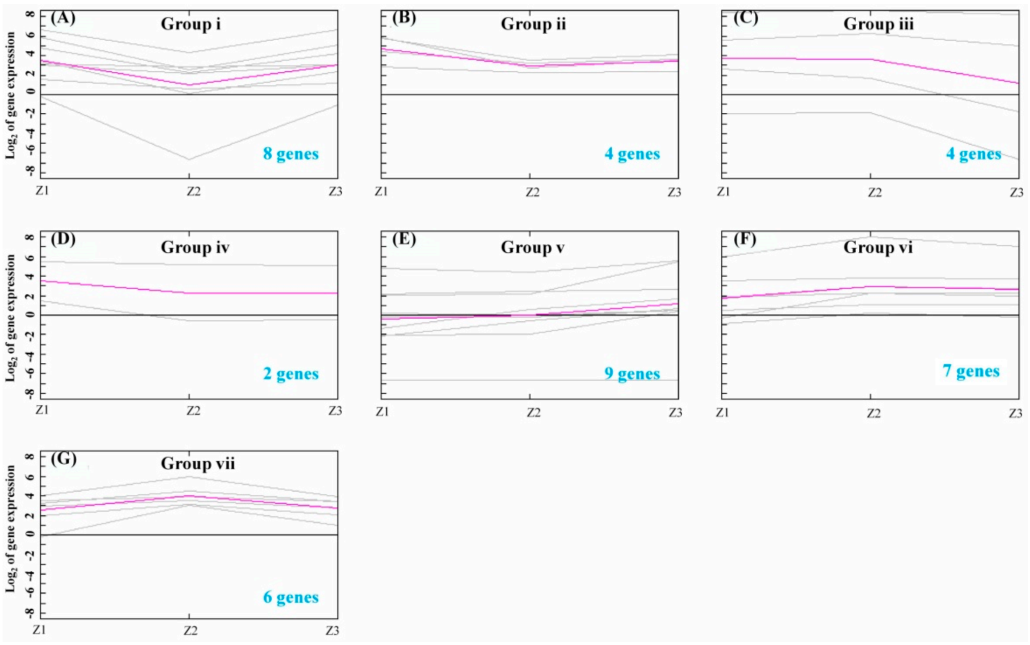

Figure S3. Expression patterns of 40 *R. glutinosa* MYB family genes in tuberous roots at different stages. (**A**–**G**) Seven expression patterns were identified to represent genes up- or down-regulated at Z1, Z2, and Z3 stage. Gene expression profiles of individual genes are depicted in gray lines, and average expression profiles for each pattern are depicted in pink lines. The line charts were generated by MultiExperiment Viewer (MeV).

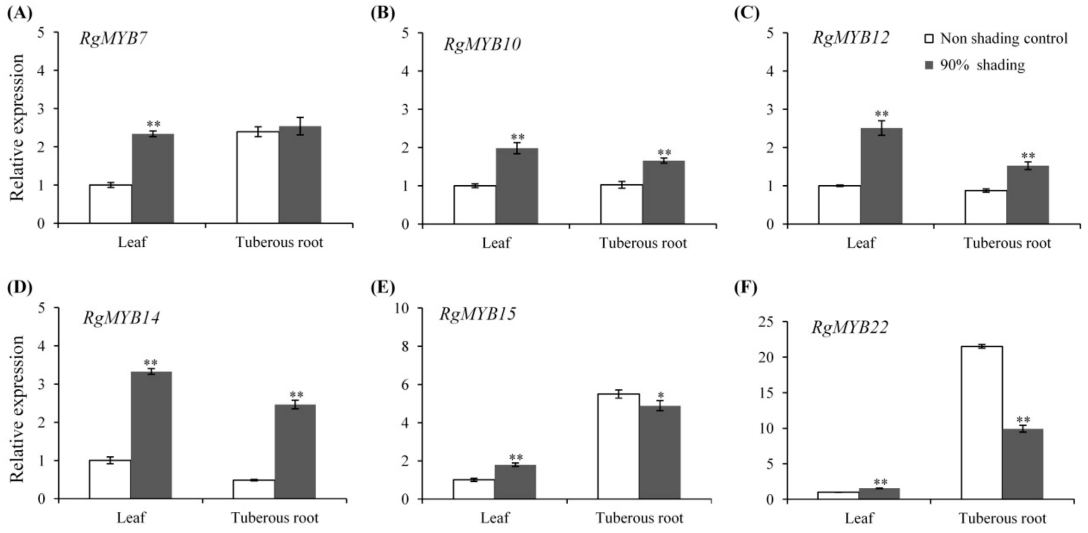

Figure S4. Quantitative RT-PCR analysis of transcript abundance of MYB family genes in leaf and tuberous root of *R. glutinosa* under 90% shading. (**A**–**F**) The expression levels of *RgMYB7* (**A**); *RgMYB10* (**B**); *RgMYB12* (**C**); *RgMYB14* (**D**); *RgMYB15* (**E**); and *RgMYB22* (**F**) under shading and non-shading conditions. The relative expression level of genes in control leaves were used as references. Mean values from three independent experiments are shown. Each independent experiment was performed with three technical replicates. Error bars represent standard deviations. For statistical analysis, 2-tailed *t*-test was performed to determine the significance of difference. * and ** indicate significant difference at $p < 0.05$ and $p < 0.01$, respectively.

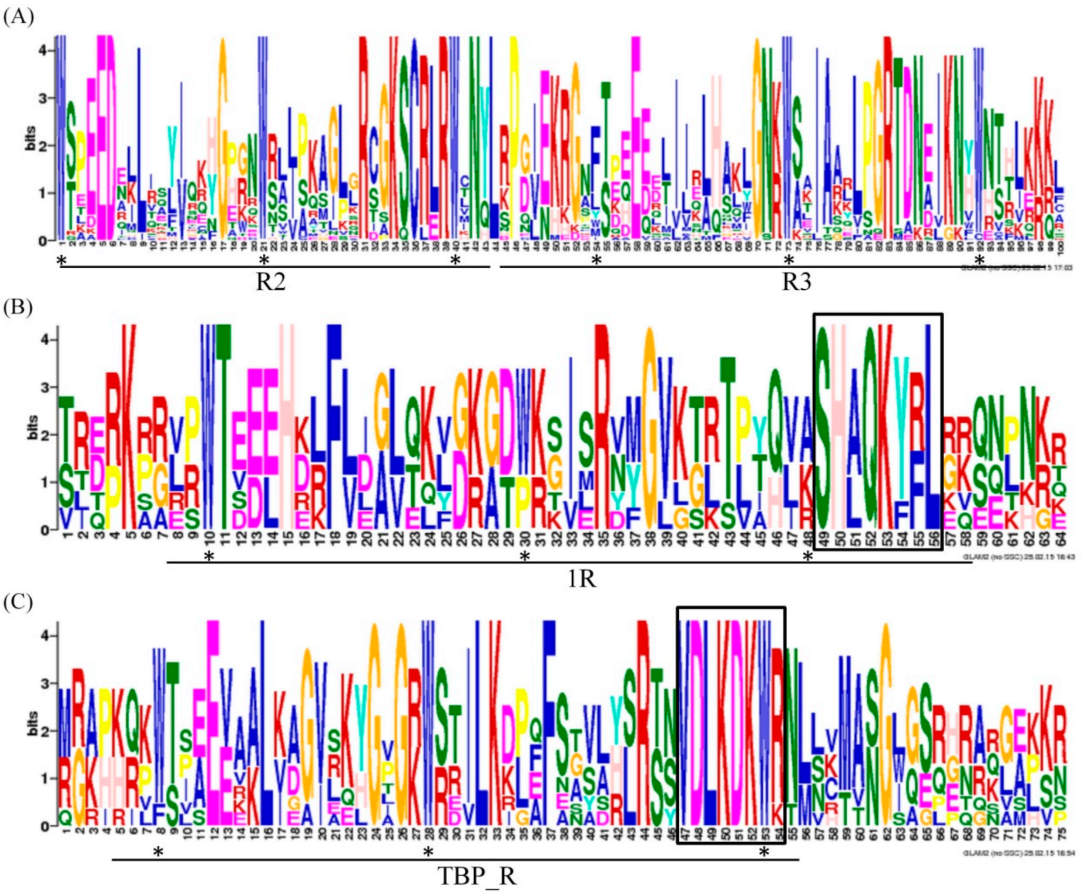

Figure S5. Conserved sequences of *R. glutinosa* MYB DNA binding domain (DBD). (**A**–**C**) Sequence logos of the 25 MYBs with R2R3 domains (**A**); 6 MYBs with 1R domains (**B**) and 8 MYBs with TRB domains (**C**). The bit score indicates the information content for each position in the sequence. Asterisks indicate the conserved tryptophan residues (Trp) in the MYB domain. The width of the amino acid positions indicates the probability of insertions (wider) and deletions (narrower). The highly conserved SHAQKYF motifs in 1R and VDLKDKWR motif in TBP_R are boxed. Logos were generated using the method described by Frith *et al.*, (2008), available at GLAM2 [64].
